# *Salmonella* Typhimurium and *Salmonella* Sofia: Growth in and Persistence on Eggs under Production and Retail Conditions

**DOI:** 10.1155/2015/914987

**Published:** 2015-10-11

**Authors:** Catherine M. McAuley, Lesley L. Duffy, Nela Subasinghe, Geoff Hogg, John Coventry, Narelle Fegan

**Affiliations:** ^1^CSIRO Food and Nutrition, Private Bag 16, Werribee, VIC 3032, Australia; ^2^CSIRO Food and Nutrition, P.O. Box 745, Archerfield BC, QLD 4108, Australia; ^3^Microbiological Diagnostic Unit, Public Health Laboratory, Department of Microbiology and Immunology, The University of Melbourne, Peter Doherty Institute for Infection and Immunity, Parkville, VIC 3010, Australia

## Abstract

Salmonellosis in Australia has been linked to eggs and egg products with specific serotypes associated with outbreaks. We compared attachment to and survival on egg shells and growth in eggs of two *Salmonella* serotypes, an egg outbreak associated *Salmonella* Typhimurium and a non-egg-associated *Salmonella enterica* ssp. II 1,4,12,27:b:[e,n,x] (*S*. Sofia). Experiments were conducted at combinations of 4, 15, 22, 37 and 42°C. No significant differences occurred between the serotypes in maximum growth rates, which were significantly greater (*P* < 0.001) in egg yolk (0.427 log_10_ CFU/mL/h) compared to whole egg (0.312 log_10_ CFU/mL/h) and egg white (0.029 log_10_ CFU/mL/h). Attachment to egg shells varied by time (1 or 20 min) and temperature (4, 22 and 42°C), with *S*. Typhimurium isolates attaching at higher levels (*P* < 0.05) than *S*. Sofia after 1 min at 4°C and *S. *Typhimurium ATCC 14028 attaching at higher (*P* < 0.05) levels at 22°C. Survival on egg shells was not significantly different across isolates. *Salmonella* serotypes behaved similarly regarding growth in egg contents, attachment to egg shells and survival on eggs, indicating that other factors more likely contributed to reasons for *S. *Typhimurium being implicated in multiple egg-associated outbreaks.

## 1. Introduction

For organisms to contaminate products or surfaces along the food chain, they must first attach. This initial attachment may take the form of loosely or firmly attached cells. Loosely attached cells are readily removed but firmly attached cells are more difficult to remove and may eventually, under the right conditions, grow to form biofilms, usually comprising a microflora of mixed populations closely associated with organic matter, assisting in the persistence of pathogenic bacteria. There are a number of potential contamination points during egg production and processing. For example, in countries where* Salmonella enterica* subsp.* enterica* serotype Enteritidis (SE) is found, infection of the laying hen may lead to internal contamination of eggs prior to oviposition (vertical transmission) or alternatively eggs may become contaminated with* Salmonella* immediately following lay, due to contact with feces or fecally contaminated laying material (horizontal transmission) [1]. The process of washing and grading eggs may also contaminate eggs via cross contamination from other eggs, contact with contaminated surfaces, or through contaminated wash water. Further contamination may occur at the consumer end of the food chain due to cross contamination from other products during storage, handling, and food preparation.

In Canada, the USA, and the EU, foodborne outbreaks due to* Salmonella* contamination of eggs are largely due to SE [2]. In Australia, eggs are predominantly contaminated with* Salmonella* Typhimurium (STM) [2]. With the predominance of SE in eggs in other parts of the world, much of the focus of international research into how* Salmonella* can be transmitted by eggs has focused on SE. The survival of* Salmonella* on the surface of eggs and subsequent penetration into the egg show a range of variable results as described in previous reviews [1, 3, 4] again with a greater focus on SE.

The predominant* Salmonella* serotypes detected in* Salmonella* infections in Australia can vary between the states and territories [5]. Numerous outbreaks of salmonellosis associated with eggs and egg products have been caused by specific, recurring serovars and phage types of* Salmonella*, including* S*. Typhimurium phage type 135 (PT135) between 2006 and 2010 [5, 6]. In particular, this phage type has been attributed to multiple salmonellosis outbreaks associated with egg consumption in Tasmania from 2005 to 2008 [7] and more recently was identified as the cause of an egg-associated salmonellosis outbreak in Canberra in 2012 [8]. A draft genome sequence of isolates associated with the Tasmanian outbreak as well as the short-term microevolution of these isolates has been published [9, 10].

This study utilized a known egg-related outbreak strain of* S*. Typhimurium PT135, a laboratory reference strain of *S*. Typhimurium, and a nonegg-associated, meat chicken-specific serovar of* S*. Sofia to test the hypothesis that an egg outbreak-associated* Salmonella* strain is better at attaching to, growing in, and/or surviving on eggs than nonegg-associated* Salmonella*. Experiments were conducted to determine attachment, survival on egg shell, and growth in egg contents in experiments at various temperatures relevant to egg production. These included 4°C, a common refrigeration temperature, 15°C, which is a production storage temperature recommended by the Australian Egg Corporation Limited [11], 22°C, ambient temperature, 37°C, which is an optimum growth temperature for* Salmonella,* and 42°C, which is the body temperature of chickens and is within the recommended egg wash temperature range [11].

## 2. Materials and Methods

### 2.1. Egg Source

Extra large eggs produced by caged hens were sourced from either local supermarkets, a single farm through farm gate sale or by arrangement with a central processor, and were stored at 4°C until use. Supermarket and farm gate sale eggs were washed and used in the growth and attachment studies. Eggs used for survival on egg shell studies were unwashed and sourced from a single farm by arrangement with a central processor.

### 2.2. Bacterial Strains

The three* Salmonella* strains assessed in this study were* S*. Typhimurium PT135 (STM), an egg outbreak strain isolated from a clinical sample, the reference strain* S*. Typhimurium ATCC 14028 (RS), and *S*. Sofia 1296a (SS) isolated from a meat chicken carcass. All three strains were used to assess the growth in and attachment to eggs. The STM and SS strains were used to assess the survival of* Salmonella* on eggs under potential consumer storage and retail conditions.

### 2.3. Growth in Eggs

The growth or survival of the* Salmonella* isolates was assessed individually in egg yolk, egg white, and whole egg. Each egg component was tested at 15, 22, and 37°C. Egg yolk and whole egg trials were conducted until the cultures reached stationary phase or up to 44 days. Counts of* Salmonella* in egg whites were followed for up to 35 days. All experiments were performed at least in duplicate.

The outside of the eggs was sanitized prior to cracking by immersion in 80% ethanol for two minutes followed by drying via evaporation in a laminar flow cabinet. Eggs were aseptically cracked and separated and egg components tipped into sterile 50 mL Falcon tubes (PLP, Blackburn, Victoria, Australia). Each egg yolk trial used two egg yolks. The egg white and whole egg trials utilized the components from one egg each. Egg whites were gently mixed using a sterile whisk to break up the egg white prior to the trials. Whole eggs were also gently mixed with a sterile whisk prior to use.

*Salmonella* strains were grown in Tryptone Soya Broth (Oxoid, Basingstoke, England) at 37°C for 20 h and then diluted in Maximum Recovery Diluent (Oxoid) so that the addition of 0.1 mL of inoculum to the egg resulted in approximately 5 × 10^2^ CFU/mL of egg content, determined by pre-trial inoculum studies, for the egg yolk and whole egg preparations at all three temperatures. The 10^2^ CFU/mL inoculum was selected as it is close to the limit of detection on 0.1 mL spread plates. The egg white treatments had a starting inoculum of approximately 5 × 10^4^ CFU/mL at all three temperatures with the egg white treatment at 15°C also tested at the lower inoculum level of 5 × 10^2^ CFU/mL. A higher inoculum level was used for the egg whites so that both growth and death could be monitored. The inoculum was mixed with the egg contents by vortexing. Uninoculated egg contents were used as the controls.

All samples were tested after inoculation (T0) and then at various intervals depending on the temperature and egg component. At each sampling period, the tubes were gently mixed by inversion 25 times. Serial dilutions of 0.2 mL were made in 1.8 mL of Buffered Peptone Water (BPW; Oxoid) and spread plated (0.1 mL) in duplicate onto Tryptone Soya Agar (TSA; Oxoid). An enrichment of 0.2 mL sample in 1.8 mL BPW was also performed when the counts fell below the limit of detection on the plates (10 CFU/mL). The limit of detection in the broth was 5 CFU/mL. All plates and enrichments were incubated at 37°C for 24 ± 2 h and then counted. Enrichments were streaked onto TSA and incubated at 37°C for 24 ± 2 h. After counting, three colonies were selected from the TSA plates of each isolate at the beginning and end of each trial and confirmed as* Salmonella* using a* Salmonella* latex test kit (Oxoid).

### 2.4. Attachment to Egg Shells

The attachment of* Salmonella* to egg shells immediately following exposure for either one or 20 min was assessed on eggs sourced from a retail outlet as well as from a single farm through farm gate sale at 4, 22, and 42°C. Eggs were equilibrated at the test temperature for a minimum of 2 h before inoculation. The inoculum was prepared by growing the strains individually in 1 L of BPW at 37°C for 18 ± 2 h without shaking. The BPW was centrifuged at 4500 ×g for 25 min at 4°C and cells were resuspended in 1 L of phosphate buffered saline (PBS; Sigma, Australia) that had been previously held overnight at 4, 22, or 42°C, as required. Eggs were dipped in the inoculum (8.66 ± 0.16 CFU/mL) for either one or 20 min, removed, and rinsed twice by gentle swirling for 5 s in 150 mL PBS equilibrated to the same temperature as used in the attachment assay. Individual eggs were then placed in a stomacher bag (Sarstedt, Australia) and massaged in the bag in 50 mL of BPW for 3 min to detach the bacteria. Each experiment was conducted in duplicate and three eggs were used for each attachment assay.

Enumeration of the* Salmonella* was conducted by spread plating 0.1 mL of suitable serial dilutions onto TSA. Plates were incubated at 37°C for 18 ± 2 h before the number of colonies was counted and recorded. A total of 10 colonies were confirmed as* Salmonella* from TSA plates using a* Salmonella* latex test kit (Oxoid). A total of three eggs from each tray were used as control eggs. These eggs were placed individually in stomacher bags and gently but vigorously rubbed and shaken in 50 mL of BPW. A portion of BPW was spread plated onto TSA and incubated at 30°C for 72 h to obtain a total viable count (TVC). Control eggs were also examined for the presence of* Salmonella* by plating onto xylose lysine deoxycholate (XLD, Oxoid) agar and enriching the remaining BPW at 37°C for 18 ± 2 h. A 0.1 mL portion was inoculated into Rappaport-Vassiliadis Soya Peptone Broth (RVS, Biomerieux, France) and incubated for 24 h at 42°C. The RVS was streaked onto XLD and incubated at 37°C for 24 h. XLD plates were examined for typical* Salmonella* colonies.

### 2.5. Survival on Egg Shells

The STM and SS strains were assessed for survival on egg shells under retail conditions at 4 and 22°C stored in clean, unused, half-dozen cardboard egg containers wrapped in brown paper. The relative humidity (RH) inside the cartons of six eggs ranged from 88 to 100% at 4°C and from 38 to 55% at 22°C.

Cultures of STM and SS were grown in Heart Infusion Broth (Oxoid) for 24 h at 37°C and viable counts determined on pour plates (1 mL) of Yeast Extract Agar [12] followed by incubation for 48 h at 37°C. Overnight broth cultures were diluted 1 : 100 in BPW prewarmed at 42°C and held at this temperature for 30 min, prior to pipetting two × 50 *μ*L (approximately 6 log_10_ CFU) aliquots to two × 1 cm^2^ areas (delineated by marker pen ink) on the surface of a prewarmed (42°C) unwashed egg, placed on a sterile wire mesh rack inside a Class II Biological Safety Cabinet and allowed to dry for up to 3 h. After the surface inoculums had dried, the uninoculated area of the eggs outside the delineated areas was swabbed with 70% ethanol and allowed to evaporate until dry so as to reduce the level of naturally occurring microflora outside of this area.

Over the duration of the four-week storage trial, five eggs were tested at week 0 (T0), then after 1, 2, and 4 weeks. Survival of* Salmonella* on individual eggs was determined by placing each egg in a sterile stomacher bag containing 10 mL BPW and held at 4°C for 16 h to soften cuticle material and then subjected to abrasion with a sterile swab to assist in removing bacterial cells from the egg surface. The BPW was then dispensed as a five tube Most Probable Number (MPN) over a 5 log_10_ dilution range using a replicate of five eggs. The remainder of the BPW not used in determination of MPN was made up to 10 mL and used to enrich the intact egg to detect surviving* Salmonella* that may have remained attached to the egg. The BPW MPN tubes and whole egg BPW enrichments were incubated for 24 h at 37°C. Following incubation, 0.1 mL was placed into 10 mL RVS (BBL Phytone Peptone, BD, Maryland, USA) and incubated for 24 h at 42°C. RVS broths were then streaked onto XLD agar and incubated for 24 h at 37°C. Colonies with typical* Salmonella* morphology on XLD were subjected to biochemical, serological agglutination and phage-typing analyses to confirm respective isolates as SS or STM. MPN values were calculated for both visible turbidity (indicative of a TVC of aerobic microorganisms) and* Salmonella*, scored using a three dilution range of five tubes. Uninoculated eggs were swabbed with 70% ethanol and treated as above for use as controls.

### 2.6. Statistical Analysis

All counts were transformed to log_10_ CFU/mL prior to statistical analysis. A line of best fit was constructed from Microsoft Excel scatter plots using a polynomial equation for the growth in egg total counts of each isolate. The DMFit predictive models (http://www.combase.cc/index.php/en/resources) were used to analyse the log_10_ CFU/mL counts for the growth in egg data sets and generate the maximum rate (CFU/mL/h). Analysis of variance (ANOVA) was conducted on the maximum rate and survival on egg shell storage trial data (time, temperature, and strain) using GenStat 13.1 (VSN International Ltd., Hemel, UK). The mean log_10_ CFU/mL count of each of six eggs (three eggs in duplicate) was used to compare attachment levels between strains, temperature, time, and source of egg (retail or farm gate) using Minitab 16 (Minitab Inc., Minnesota, USA). A one-way analysis of means using Tukey's method for multiple comparisons was performed on pairs of data sets using Minitab. Results were considered significant at *P* < 0.05.

## 3. Results

### 3.1. Growth in Eggs

The growth rates of STM, SS, and RS were not significantly different (*P* > 0.05) in any of the egg components at any temperature, showing that the outbreak strain of* Salmonella* Typhimurium did not grow faster than the other* Salmonella* isolates and suggesting that multiple* Salmonella* species could grow quickly in egg contents if conditions were suitable. As the growth rates between the isolates were not significantly different (*P* > 0.05), the results were pooled for each egg component and temperature. The mean maximum growth rates of* Salmonella* were determined in each of the egg components at different temperatures (Table 1) with the greatest growth rates occurring at higher temperatures for each egg component. Growth was significantly greater (*P* < 0.001) in the egg yolk, than in the whole egg or egg white at all temperatures. The highest maximum growth rates occurred in egg yolks at 37°C and ranged from 0.7735 to 0.9064 log_10_ CFU/mL/h. The lowest maximum growth rates occurred in the egg whites with negative growth rates (ranging from −0.0138 to 0.0187 log_10_ CFU/mL/h) observed in egg whites incubated at 15°C. Inoculation level and isolate did not play a role in determining whether* Salmonella* counts increased or decreased in the egg white at 15°C. At 22°C in the egg white, the majority of growth rates were positive and ranged from −0.005 to 0.1015 log_10_ CFU/mL/h. Then at 37°C in the egg white, the majority of growth rates were again positive but ranged from −0.1227 to 0.1434 log_10_ CFU/mL/h.

All of the* Salmonella* isolates grew to stationary phase (10^8^-10^9^ CFU/mL) in the egg yolk and whole egg. Stationary phase was only achieved in six of the 24 higher starting inoculum egg white experiments. The time to reach stationary phase in the egg yolk was three days at 15°C, 26 h at 22°C, and 9 to 10 h at 37°C for each of the strains. In the whole egg stationary phase was reached in four days at 15°C, 34 h at 22°C, and 12 to 16 h at 37°C. The isolates did not grow in the egg white compared to the yolk and whole egg as well and there was more variation between replications in the egg white. The survival of* Salmonella* in egg white was tested at two inoculum levels at 15°C. The largest maximum standard deviation between replicate time points was seen in the egg white at 15°C at the higher inoculum for STM. In the high inoculum egg white at 15°C, only one isolate (STM) reached 10^8^ CFU/mL after 34 days in one replicate experiment, whereas the numbers of all of the other replicates and isolates either remained steady or declined over time. All of the isolates decreased in number in egg white when the lower inoculum was used and dropped to near or below the limit of detection after 35 days. At 22°C, the maximum standard deviations between replicate time points were 1.5- to 3-fold greater in the egg white than in the yolk and whole egg for all three strains. Reproducibility between trials in egg white at 22°C was variable with the time to grow to stationary phase different between trials and between strains, ranging from three to 30 days or longer. At 37°C, the level of* Salmonella* in egg white remained relatively unchanged for 16 h, but after six days the counts of STM had dropped by one log_10_ and the viable count for RS had dropped below the limit of detection (5 CFU/mL), while SS counts remained unchanged.* Salmonella* was not detected in the control eggs.

### 3.2. Attachment to Eggs

No* Salmonella* was detected on control eggs. All TVC were <410 CFU/mL. All* Salmonella* isolates attached to eggs at similar levels with the highest attachment (5.85 log_10_ CFU/mL) by RS at 22°C for 20 min on farm gate sourced eggs, while the lowest level of attachment (3.77 log_10_ CFU/mL) was by SS at 4°C for 1 min on farm gate sourced eggs (Tables 2 and 3). STM and SS attached at significantly (*P* < 0.05) lower levels than RS at 22°C on farm eggs. STM and RS attached at significantly (*P* < 0.05) higher levels than SS after 1 min at 4°C on farm eggs.

Within all combinations of strain, egg source, and temperature, the attachment levels after 1 min were lower than after 20 min. However, this difference was significant (*P* < 0.05) only for strains SS and RS when attached to farm eggs at 22°C. Strain differences were noted only on farm sourced eggs for a number of combinations of time/temperature. At 22°C STM attached significantly lower than RS on farm eggs, but not at a significantly (*P* > 0.05) different level to SS after 20 min. STM and RS attached at significantly (*P* < 0.05) higher levels after 1 min than SS on farm eggs at 4°C. There was a significantly (*P* < 0.05) lower level of attachment of SS after 20 min compared to RS at all temperatures. The source of eggs either retail or farm did not affect the level of attachment of either STM or RS for any time/temperature combination. However the source of eggs, retail (5.26 log_10_ CFU/mL) or farm gate (4.68 log_10_ CFU/mL), had a significant (*P* < 0.05) effect on the level of attachment of SS at 42°C after 1 min.

Temperature also had an interactive effect on the level of attachment of some strain, time, and egg source combinations. On retail eggs at 4°C, SS attached at a significantly (*P* < 0.05) lower level than at 42°C after 1 min of attachment time. On farm gate sourced eggs, SS at 4°C again attached at significantly (*P* < 0.05) lower levels than at 42°C, after 1 min but not after 20 min.* Salmonella* was not detected on any control eggs.

### 3.3. Survival on Eggs

The TVC of both eggs inoculated with* Salmonella* and the uninoculated control eggs stored at 4°C showed a decreasing trend in the first two weeks of storage with an approximately 2.0 log_10_ MPN/cm decrease after two weeks. In contrast, eggs stored at 22°C challenged with SS maintained viable count levels throughout four-week storage. At the same temperature, eggs challenged with STM showed a similar trend, although a decrease of about 1.0 log_10_ MPN/cm was observed at week four.

The results for recovery of* Salmonella* from egg surfaces indicate that neither strain of SS or STM persisted past four weeks. SS was recovered from eggs by either MPN or selective enrichment procedures applied to intact eggshells and was isolated at both storage conditions at week two (four out of five eggs) whereas STM could only be recovered after one week of storage (two out of five eggs) at 22°C. At 4°C, STM was not detected on any of the five eggs after one week. Evaluating the viable level of* Salmonella* recovered using an MPN procedure also showed that there was rapid decline in the survival of* Salmonella* after one week of storage (Table 4). The surviving* Salmonella* count was minimal at 22°C. At 4°C the mean count of SS was 33 MPN/cm (1.52 log_10_) and 3 MPN/cm (0.48 log_10_) at weeks one and two, respectively, compared with <0.9 MPN/cm (<−0.05 log_10_) for STM. None of the* Salmonella* strains survived after four-week storage under either refrigerated or ambient conditions. The overall trend in log_10_ reduction of the* Salmonella* count was similar for both strains.

## 4. Discussion

As anticipated, all of the* Salmonella* isolates reached stationary phase rapidly in egg yolk when incubated at 37°C. The growth rate was not different between SS, RS, and the egg-related outbreak strain STM in any egg component, regardless of temperature. The nutrient-rich environment in the egg yolk allows the quick growth of SE [13] and this would not be expected to be different for other* Salmonella* serovars. Even at 15°C, stationary phase was reached after three days in the egg yolk and four days in the whole egg for all of the isolates in this study. This indicates that if* Salmonella* were to contaminate eggs and access the egg yolk,* Salmonella* numbers would be very high before the best before dates of eggs were reached, even with the eggs stored at the recommended AECL storage temperature of 15°C [11]. In a study by Cogan and others [14]* Salmonella* levels of >10^6^ CFU/mL were used to indicate that SE and STM had been able to invade the egg yolk after inoculation in the albumen. These authors concluded that motility of the isolates was necessary for this to occur and that the presence of curli fimbriae permitted greater growth of the* Salmonella* [14]. Both SS and RS have the presence of curli fimbriae [15], although this is undetermined for STM. It is unknown whether or not the presence of fimbriae may have resulted in the varied growth rates in egg white in this study and is worthy of further investigation.

Musgrove et al. [16] looked at the growth of a two strain cocktail of STM DT104 at 4, 10, 20, 30, 37, and 42°C in pasteurized liquid whole egg and egg white, although the trials did not extend beyond 16 days. STM grew at all temperatures in whole egg except at 4°C and also grew in egg white from 20 to 37°C, but not at 4, 10, or 42°C [16]. The STM isolates in the current work, as well as the* Salmonella* Sofia, appeared to reach stationary phase more quickly at 37°C than the STM DT104 isolates (12–16 h versus 20 h, resp.), even with a lower starting inoculum. That study used an initial inoculum of 10^4^ CFU/mL [16] which would be considered a high starting inoculum given that eggs naturally contaminated with* Salmonella* would generally have <20 CFU/egg [17]. If a lower starting inoculum was used, the time to reach stationary phase in egg yolk or a whole egg would increase. This would also increase the time before an infectious dose was reached. The infectious dose of* Salmonella* spp. is considered to be 10^2^-10^3^ CFU/mL [18]. However, it would only take the isolates in this study two days at 15°C and 20 h at 22°C to reach an infectious dose in a whole egg if starting with a count of 20 CFU/egg. In order to prevent the growth of* Salmonella* in eggs, it is critical that eggs can be stored at temperatures that inhibit the growth of* Salmonella*, which is below 7°C for most salmonellae [19].

In the current study, attachment levels were always lower after 1 min compared to 20 min although only one combination was significant. The level of attachment of SS was significantly lower at 4°C compared to 42°C after 1 min. As the Code of Practice for shell eggs states that the wash water should be between 41 and 44°C, any* Salmonella* that may contaminate the wash water may attach at a higher level than if lower temperatures were used. This suggests that removal of gross faecal contamination as soon as possible after laying may assist in the reduction of the level of attached* Salmonella*. High levels of initial attachment have been noted for other organisms with rapid attachment within 1 min recorded for* Campylobacter* on stainless steel [20] and* Listeria* and* Pseudomonas* on raw potato tissue [21]. Temperature has also been shown to affect the level of attachment of* Campylobacter* and* Listeria* to stainless steel, with increasing levels of attachment noted with increasing temperature [20, 22], which was also seen when comparing SS on retail eggs at 4 and 42°C in contrast to other strains and conditions used in this study.

*S*. Sofia strain S1296a has been previously found to attach to five abiotic surfaces at significantly higher levels than *S*. Typhimurium strains, including the ATCC 14028 strain (RS) used in this study [23]. SS attached in significantly lower levels than RS after a period of 20 min attachment, but only on farm eggs. Properties of both the cell and the substrate are known to affect the attachment of bacteria. Surface properties such as surface roughness, surface free energy, and hydrophobicity may affect bacterial attachment and are reviewed by Goulter et al. [24]. STM, an egg outbreak strain, attached at similar levels to the SS, at 22°C with both strains attaching at significantly lower levels than RS. However at 4°C STM attached at levels similar to the other* S*. Typhimurium strain, RS. Attachment is a complex process and the limited number of strains and conditions used in this study along with the level of variation recorded make it difficult to ascertain precise temperatures for minimizing attachment. Further studies should examine both the surface of the egg and an increased number of strains.

One of the observations in the current investigation is the variability in recovery of* Salmonella* from egg surfaces. Other studies [25, 26] show similar variability in the recovery of* Salmonella* from egg surfaces and a rapid decline in viable numbers of SE adsorbed to egg surfaces. In the current study, egg surfaces inoculated with* Salmonella* were stored under refrigeration with high RH and ambient room temperature storage conditions that may occur in Australian consumers' households. The survival of SS after drying on the surface of eggs was similar to that of STM, with the only significant difference (*P* < 0.01) occurring after one week of storage where SS counts were higher at 4°C than at 22°C and higher than STM at both temperatures. In addition, SS was still at countable levels on egg shells after two-week storage, suggesting that SS may have an advantage in survival and particularly under refrigeration conditions. This is contrary to published literature which suggests that the greater survival [26] of* Salmonella* at 22°C rather than 4°C after seven days storage is likely related to high humidity under refrigerated conditions compared to ambient temperature. In Australia, SS is the dominant* Salmonella* serotype on chicken carcasses both before and after processing [27]. These results may indicate that SS can endure certain stresses that occur in the final, chilled stages of poultry processing better than other* Salmonella* serovars in Australia.

## 5. Conclusion

This study aimed to determine if the egg outbreak-related strain of *S*. Typhimurium (STM) grew in or attached and survived on egg shells in higher levels than a strain of *S*. Sofia (SS) (not associated with eggs) and a reference strain of* Salmonella* Typhimurium (RS).* Salmonella* egg outbreaks in Australia are associated with contamination on the egg, not due to organisms internalized during the formation of the egg; thus information on the attachment of a* Salmonella* outbreak strain on egg shells is new and valuable for understanding how to limit contamination of eggs with* Salmonella*. Differences in the growth of these strains in egg contents were not observed, with all of the isolates growing rapidly to stationary phase in egg yolk and whole egg, but not egg white. A first step in the contamination of eggs with* Salmonella* is the attachment of* Salmonella* to the egg shell. In this study, the egg outbreak-associated STM was not able to attach to eggs in significantly higher levels than the other strains and it is likely that factors other than attachment to egg surfaces contributed to the reasons that this particular serotype caused an outbreak associated with eggs. Furthermore, the SS and STM strains exhibited similar survival on the surface of the eggs and survival was minimal after two weeks and not detected after four weeks. Bacterial survival on egg surfaces decreased under storage at refrigerated temperature (4°C) associated with high humidity (>88% RH) more than under ambient conditions of temperature and humidity. Notwithstanding the need to maintain hygienic egg harvesting, washing and distribution handling practices, it is critical that eggs can be stored at temperatures less than 7°C, which is the accepted minimum temperature for growth of* Salmonella* in food. Existing industry practices of maintaining a retail product shelf-life of six weeks would be best undertaken by maintaining refrigeration conditions, desirably at 4°C as indicated in the current study, so as to limit the risk of survival of* Salmonella* and other microflora present on eggs.

## Figures and Tables

**Table 1:** Mean maximum growth rates (CFU/mL/h) of *Salmonella* isolates with the higher inoculum level in the egg white.

Temperature	SS^1^			STM^2^			RS^3^			All isolates combined^4^		
	Egg yolk	Whole egg	Egg white	Egg yolk	Whole egg	Egg white	Egg yolk	Whole egg	Egg white	Egg yolk	Whole egg	Egg white
15°C	0.116	0.093	0.005	0.115	0.099	−0.007	0.112	0.094	−0.001	0.114^aA^	0.095^aA^	−0.001^bA^
22°C	0.324	0.211	0.022	0.323	0.238	0.023	0.328	0.237	0.040	0.325^aB^	0.228^bB^	0.028^cAB^
37°C	0.821	0.613	0.095	0.865	0.614	0.076	0.840	0.610	0.010	0.842^aC^	0.612^bC^	0.060^cB^
All temperatures	0.420	0.306	0.041	0.427	0.314	0.016	0.434	0.317	0.031	0.427^a^	0.312^b^	0.029^c^

^1^SS: *S*. Sofia 1296a; ^2^STM: *S. *Typhimurium PT135; ^3^RS: *S*. Typhimurium ATCC 14028.

^4^There were no significant differences between the isolates individually (*P* > 0.05); therefore the isolates were combined to assess the effects of temperature and egg component. Different lower case letters within rows are significantly different (*P* < 0.001). Different upper case letters within columns are significantly different (*P* < 0.001).

**Table 2:** The level of attachment of each isolate for retail eggs (± standard deviation) at 4, 22, and 42°C after 1 or 20 min of attachment.

Temperature	SS^1^		STM^2^		RS^3^	
	1 min	20 min	1 min	20 min	1 min	20 min
4°C	4.43 ± 0.36^aA^	4.78 ± 0.29^abA^	5.01 ± 0.08^abA^	5.14 ± 0.34^bA^	4.96 ± 0.31^abA^	5.36 ± 0.24^bA^
22°C	4.78 ± 0.80^aAB^	5.24 ± 0.18^aA^	4.88 ± 0.35^aA^	5.19 ± 0.23^aA^	4.99 ± 0.23^aA^	5.25 ± 0.46^aA^
42°C	5.26 ± 0.28^aB^	5.32 ± 0.15^aA^	4.82 ± 0.26^aA^	5.1 ± 0.15^aA^	4.85 ± 0.28^aA^	5.28 ± 0.42^aA^

Different lower case letters within rows are significantly different (*P* < 0.05). Different upper case letters within columns are significantly different (*P* < 0.05).

^1^SS: *S*. Sofia 1296a; ^2^STM: *S. *Typhimurium PT135; ^3^RS: *S.* Typhimurium ATCC 14028.

**Table 3:** The level of attachment of each isolate for farm eggs (± standard deviation) at 4, 22, and 42°C after 1 or 20 min of attachment.

Temperature	SS^1^		STM^2^		RS^3^	
	1 min	20 min	1 min	20 min	1 min	20 min
4°C	4.14 ± 0.25^aA^	4.64 ± 0.19^abA^	4.72 ± 0.39^bcA^	5.11 ± 0.15^bcdA^	5.10 ± 0.16^bcdA^	5.23 ± 0.16^cdeA^
22°C	4.16 ± 0.18^aAB^	4.85 ± 0.12^bcA^	4.60 ± 0.19^abA^	5.00 ± 0.20^bcA^	4.83 ± 0.14^bcA^	5.75 ± 0.10^dA^
42°C	4.68 ± 0.19^aB^	4.81 ± 0.24^abA^	5.06 ± 0.65^abcA^	5.09 ± 0.26^abcA^	5.04 ± 0.12^abcA^	5.56 ± 0.21^cA^

Different lower case letters within rows are significantly different (*P* < 0.05). Different upper case letters within columns are significantly different (*P* < 0.05).

^1^SS: *S*. Sofia 1296a; ^2^STM: *S*. Typhimurium PT135; ^3^RS: *S.* Typhimurium ATCC 14028.

**Table 4:** Survival on egg shells during storage at 4 and 22°C.

Time (weeks)	4°C (log_10_⁡MPN/cm^2^)^1^		22°C (log_10_⁡MPN/cm^2^)	
	SS^2^	STM^3^	SS	STM
0	3.89^aA^	3.39^abA^	3.55^abA^	2.88^bA^
1	1.52^aB^	<−0.05^bB^	0.56^bB^	0.25^bB^
2	0.48^aC^	<−0.05^aB^	0.10^aB^	<−0.05^aB^
4	<−0.05^aC^	<−0.05^aB^	<−0.05^aB^	<−0.05^aB^

Different lower case letters within rows are significantly different and different upper case letters within columns are significantly different (*P* < 0.01).

^1^Mean MPN/cm^2^ of five eggs at each sampling time.

^2^SS: *S*. Sofia 1296a; ^3^STM: *S. *Typhimurium PT135.
